# Addition to the Staff

**Published:** 1913-01

**Authors:** 


### Addition to the Staff

It gives us great pleasure to announce the acceptance of the position of Associate Editor for France, by Dr. René Gaultier, of Paris. Dr. Gaultier's works, Clinical Coprology and Diseases of the Duodenum are classics. In 1908, at the Hotel Dieu, we derived great profit from his clinical and laboratory courses though hampered somewhat at the beginning because unfamiliarity with the language seemed to imply denser ignorance than was really our case. With the appointment of Dr. Gaultier, our foreign staff may be considered complete, although, at some future time, other countries will probably be represented. It may not be out of place to add that the men whose names lend dignity to the JOURNAL, have favored the editor with personal kindness and hospitality and are not merely names but living entities of happy memories and pleasant anticipation. The support, advice and cooperation of these men are deeply appreciated.

Back Numbers Wanted. Our reserve file of the following issues has been exhausted by unexpected demands: Oct. and Nov., 1911; Feb., March, July, Sept., Oct., 1912. We will give three months extension of an existing subscription, three months of a new subscription or three copies of any current issue desired for each of the back numbers supplied.

